# Glycyl-L-Prolyl-L-Glutamate Pseudotripeptides for Treatment of Alzheimer’s Disease

**DOI:** 10.3390/biom11010126

**Published:** 2021-01-19

**Authors:** Hasan Turkez, Ivana Cacciatore, Lisa Marinelli, Erika Fornasari, Mehmet Enes Aslan, Kenan Cadirci, Cigdem Yuce Kahraman, Ozge Caglar, Abdulgani Tatar, Giuseppe Di Biase, Ahmet Hacimuftuoglu, Antonio Di Stefano, Adil Mardinoglu

**Affiliations:** 1 Department of Medical Biology, Faculty of Medicine, Atatürk University, 25240 Erzurum, Turkey; 2 Department of Pharmacy, Univerisity “G. d’Annunzio” of Chieti-Pescara, Via dei Vestini 31, 66100 Chieti Scalo, Chieti, Italy; cacciatore@unich.it (I.C.); l.marinelli@unich.it (L.M.); e.fornasari@unich.it (E.F.); giuseppe.dibiase@unich.it (G.D.B.); adistefano@unich.it (A.D.S.); 3 Department of Molecular Biology and Genetics, Faculty of Science, Erzurum Technical University, 25200 Erzurum, Turkey; enesiyte@gmail.com (M.E.A.); cglrozg@gmail.com (O.C.); 4 Department of Internal Medicine, Erzurum Regional Training and Research Hospital, Health Sciences University, 25200 Erzurum, Turkey; doktorcadirci@hotmail.com; 5 Department of Medical Genetics, Faculty of Medicine, Atatürk University, 25240 Erzurum, Turkey; cigdem.kahraman@atauni.edu.tr (C.Y.K.); agtatar@gmail.com (A.T.); 6 Department of Medical Pharmacology, Faculty of Medicine, Atatürk University, 25240 Erzurum, Turkey; hacimuftuoglu@gmail.com; 7 Science for Life Laboratory, KTH—Royal Institute of Technology, 24075 Stockholm, Sweden; 8 Centre for Host-Microbiome Interactions, Dental Institute, King’s College London, London SE1 9RT, UK

**Keywords:** Alzheimer’s disease, neurotoxicity, glycine-proline-glutamate peptidomimetics, in vitro cell culture model, gene expressions

## Abstract

So far, there is no effective disease-modifying therapies for Alzheimer’s Disease (AD) in clinical practice. In this context, glycine-L-proline-L-glutamate (GPE) and its analogs may open the way for developing a novel molecule for treating neurodegenerative disorders, including AD. In turn, this study was aimed to investigate the neuroprotective potentials exerted by three novel GPE peptidomimetics (GPE1, GPE2, and GPE3) using an in vitro AD model. Anti-Alzheimer potentials were determined using a wide array of techniques, such as measurements of mitochondrial viability (MTT) and lactate dehydrogenase (LDH) release assays, determination of acetylcholinesterase (AChE), α-secretase and β-secretase activities, comparisons of total antioxidant capacity (TAC) and total oxidative status (TOS) levels, flow cytometric and microscopic detection of apoptotic and necrotic neuronal death, and investigating gene expression responses via PCR arrays involving 64 critical genes related to 10 different pathways. Our analysis showed that GPE peptidomimetics modulate oxidative stress, ACh depletion, α-secretase inactivation, apoptotic, and necrotic cell death. In vitro results suggested that treatments with novel GPE analogs might be promising therapeutic agents for treatment and/or or prevention of AD.

## 1. Introduction

**Figure 1:**
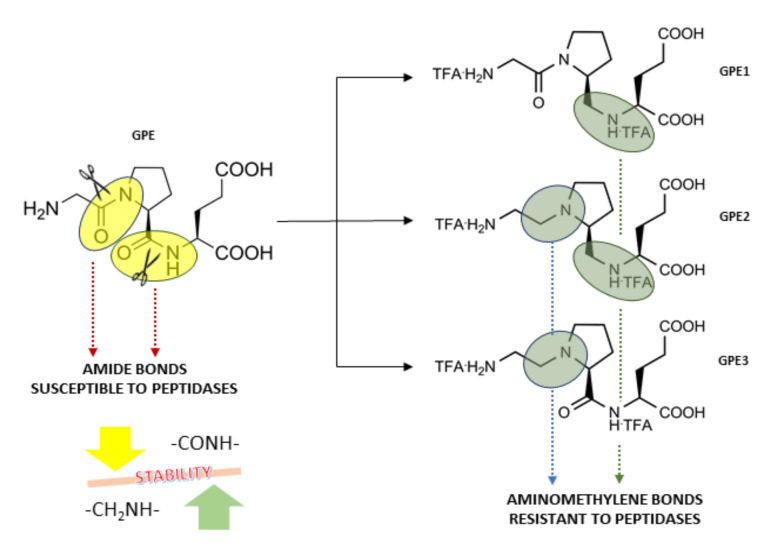
Design and development of GPEs peptidomimetics starting from native GPE.

The glycine-L-proline-L-glutamate (GPE) (Figure 1) is a naturally cleaved N-terminal tripeptide of IGF-1 via brain proteases. It is commercially available as Glypromate [1,2]. GPE executes remarkable neuroprotective properties in several in vitro and in vivo models, demonstrating its involvement in traumatic brain injury and chronic neurodegeneration [3,4,5,6,7,8,9]. Literature showed that GPE treatment has a positive impact on the proliferation and migration of mouse neural stem cells (NSCs) by altering extracellular signal-regulated kinase (ERK) and phosphoinositide 3-kinase PI3K-Akt pathways associated with neuroprotective activity [10]. Additionally, amyloid-beta (Aβ) peptide is believed to induce ROS’s excessive formation via the mechanism requiring NMDA receptor activation on hippocampal neuronal cultures. At this point, GPE has been shown to have a binding affinity toward NMDA receptors, but not to other ionotropic glutamate subfamilies, such as (2S)-2-amino-3-hydroxy-5-methyl-4-isoxazole propionic acid (AMPA) or kainate (KA) receptors. Hence, GPE is proposed to be a potential target for rational design of neuroprotective agents for neurological disorders [10]. Interestingly, phase 3 trial using Glypromate product to modulate the cognitive impairment in patients undergoing cardiac surgery with bypass was failed. However, in the last decade, variegated neuropeptides derived from IGF-I were found to exhibit adequate protection and more favorable pharmacokinetic profile compared to IGF-I [11,12].

Nonetheless, the therapeutic potentials of GPEs remain limited, owing to the role of peptidases in degradation process. Again, metabolic stability results indicated that GPE has a very short plasma half-life, and this consequence restricts its central nervous system (CNS) delivery. Therefore, development of novel neuropeptides to overcome the limitations of their endogenous counterparts would provide a fruitful drug discovery approaches for treatment of AD. To eliminate these stability issues, different strategies were applied for modification of GPE molecule, since its structural simplicity open a new route for the synthesis of sterically hindered peptides and/or non-peptide derivatives with increased bioavailability and improved metabolic stability [13].

Previously, an intimate elaboration was made for the replacement and/or modification of GPE amino acid sequence to (I) improve its proteases resistance and (II) manage and retaining its neuroprotective feature [12]. Therefore, the introduction of peptide bond surrogates has been recommended as an effective strategy to prolong the lifetime of peptides, improve the biological activity or selectivity, design enzyme inhibitors, and induce conformational features. Thus, we obtained three novel GPE peptidomimetics (GPE1, GPE2, and GPE3), with peptide bonds reduced to an aminomethylenic group at the Gly-Pro (GPE3) or Pro- Glu (GPE1), or both the junctions (GPE2) to improve the stability against proteolytic attacks still exhibiting their biological activity (Figure 1). Preliminary data showed that the introduction of the aminomethylene unit into the GPE sequence would provide stable GPE 1-3 pseudotripeptides (Figure 1). As we expected, the removal of the amidic bond with an isosteric group would increase the half-life of the three pseudotripeptides compared to that of GPE. Notably, plasma stability features showed a half-life (t_1/2_) > 4.5 h that would permit them to reach unaltered the site of action (neuroinflammed areas) in the CNS [12].

Herein, we aimed to evaluate the multi-target profile exerted by the novel GPE peptidomimetics and propose them as potential anti-Alzheimer drug candidates in AD therapy. With this design, the anti-Alzheimer evaluations were materialized with a wide range of basic techniques in the cellular AD model. These techniques, include (I) measurements of mitochondrial viability (MTT) and lactate dehydrogenase (LDH) release (II) determination of AChE, α-secretase and β-secretase activities, (III) establishment of total antioxidant capacity (TAC) and total oxidative status (TOS) levels, (IV) flow cytometric and microscopic detection of apoptotic and necrotic neuronal death, and (V) definition of molecular genetic responses via PCR arrays. 

## 2. Materials and Methods 

Synthesis of GPE1-3 was conducted by Marinelli et al. [12]. All reagents for the synthesis of compounds were procured from Sigma-Aldrich (St. Louis, MO, USA).

### 2.1. The Cellular Model of AD

The SH-SY5Y cell line (ATCC CRL-2266) was obtained from American Type Culture Collection (ATCC, Manassas, VA, USA). Cell cultures were grown in DMEM: F12 (1:1) (Gibco, Grand Island, NY, USA) with 10% fetal bovine serum (FBS) (Sigma-Aldrich, St Louis, MO, USA) and 1% penicillin/streptomycin (Sigma-Aldrich) at 37 °C and 5% CO_2_. After the cultures reached 80% confluence, the cells were detached from the flask’s surface using trypsin/EDTA (Sigma-Aldrich) solution and then seeded to 48 well plates. For the differentiation procedure, 10 μM RA (Sigma-Aldrich) was added to the media and incubated for a week. Afterward, the media were supplemented with 25 nM BDNF (Promega, Fitchburg, WI, USA) for another 5 days to complete cellular differentiation. Differentiated cells were determined under the inverted microscope (Olympus CKX41, Düsseldorf, Germany) and flow cytometric cell cycle analysis. The cells were used within 7 days [14,15,16]. Briefly, differentiated and undifferentiated cell cultures were stained with 5 µL propidium iodide (PI, 50 µg/mL), and then were incubated at room temperature in the dark for 5 min. Next, a flow cytometer (Partec, Muenster, Germany) was used to analyze the cellular DNA content frequency histograms for cell distributions in three major cycle phases (G1 vs. S vs. G2/M).

### 2.2. Treatments

Different concentrations of GPE, GPE1, GPE2, GPE3, memantine hydrochloride (MEM) (0.1, 1, 10, 25, 50, and 100 μM), and *Aβ_1-42_* (20 μM) (Sigma-Aldrich) were applied to differentiated cell culture for 24 h (n = 5). For negative control, non-treated differentiated cell culture was investigated. The tripeptide glycine-proline-glutamate abbreviated as GPE was used to compare novel peptidomimetics’ biological effects (GPE1-3). A 1% Triton-X as a nonionic detergent, ascorbic acid (10 μM) as an effective antioxidant, and H_2_O_2_ (25 µM) as an oxidizing agent (Sigma-Aldrich) were used as positive controls for cell viability, TAC, and TOS analysis, respectively. AChE activity was compared to Galantamine hydrobromide (GAL) as an anticholinesterase drug (Sigma-Aldrich). Likewise, gallic acid (GA) (Sigma-Aldrich) was used as a positive control for α-secretase and β-secretase activity assays.

### 2.3. MTT Assay

Cell proliferation was analyzed using MTT commercially available assay kits (Cayman Chemical Company, Ann Arbor, MI, USA). After the medium aspirating process of 170 μL MTT solution was added to each well. Plates were incubated for 3 h at 37 °C, and then were centrifuged at 1200 rpm for 3 min. The formazan crystals were dissolved in 200 μL dimethyl sulfoxide (DMSO) (Sigma-Aldrich). Absorbance at 570 nm was measured using a plate reader (BioTek, Winooski, VT, USA). Cell viability rates (n = 5) were calculated as percentage relative to the untreated control value [17,18].

### 2.4. LDH Assay

LDH cytotoxicity assay (Cayman Chemical Company) was performed according to the manufacturer’s recommendations. The cells were incubated in 48-well plates, and different concentrations of drugs were exposed for 24 h (n = 5). Subsequently, 100 µL supernatant and 100 µL of the reaction mixture were placed on a fresh 48-well plate and incubated for 30 min. Next, a microplate reader measured the absorbance of the samples at 490 nm [19].

### 2.5. Determination of AChE Activity 

The colorimetric kits from Abcam (Cambridge, MA, USA) were used to analyze AChE activity for the cellular AD model (n = 5) according to the manufacturer’s instructions [20].

### 2.6. Determination of β-Secretase and α-Secretase Activities

The activities of β-secretase and α-secretase enzymes in e differentiated SH-SY5Y cultures (n = 5) were measured by the commercially available fluorometric activity detection kits (Sigma-Aldrich) according to the provider’s recommended protocols [16].

### 2.7. TAC and TOS Analysis

In the TAC assay, antioxidants in samples obtained from the cellular AD model reduce dark bluish-green colored 2,2′-azino-bis (3-ethylbenzothiazoline-6-sulfonic acid) diammonium salt (ABTS) to a colorless reduced form of ABTS. The recorded change in absorbances at 660 nm is correlated with the sample’s total antioxidant capacity. TAC assay was calibrated using a vitamin E analogue, named as Trolox equivalent. In TOS assay, remaining oxidants in samples quickly oxidize the ferrous ion–chelator complex to ferric ion. The reaction medium also contains a wealth of enhancer molecules to prolong the oxidation reaction. The ferric ion produces a colored complex with chromogen. The spectrophotometrically determined color intensity is directly related to the total oxidant molecules available in the sample. The TOS assay was calibrated using H_2_O_2_, and the results are presented as µM H_2_O_2_ equivalent per liter. The automated TAC and TOS assays were performed using commercially available kits (n = 5) (Rel Assay, Gaziantep, Turkey) [21,22].

### 2.8. Apoptosis Detection by Hoechst 33258 Staining

Hoechst 33258 staining was used for morphological assessment of apoptotic cells. The untreated and treated cells were fixed with 4% paraformaldehyde in phosphate-buffered saline at 4 °C for 20 min. The cells were washed twice with PBS and then stained with 1 mM 33258 fluorescent dye (Sigma-Aldrich). Cells (n = 5) were observed and photographed under fluorescence microscopy (Leica DM IL LED, Wetzlar, Germany) [23]. 

### 2.9. Apoptosis-Necrosis Assay

The frequencies of viable, apoptotic, and necrotic cells were determined using the Annexin V-FITC apoptosis detection kit I (BD Pharmingen, Waltham, MA, USA). For each sample (n = 5), 5 × 10^4^ cells were mixed with 500 µL of 1× binding buffer. A 5 µL of Annexin V-FITC and 5 µL propidium iodide (PI, 50 µg/mL) were added to the samples and incubated at room temperature for 5 min in the dark. Finally, CyFlow Cube 6 flow cytometer (Partec) system was used to determine apoptotic and necrotic cell death ratios [24].

### 2.10. Molecular Genetic Analysis

The complete molecular genetic analyses were performed in three stages, as described below.

#### 2.10.1. Total RNA Isolation

A 5 × 10^6^ undifferentiated SHSY-5Y cells were seeded in a 6-well plate and differentiated into neuron-like cell culture via *all-trans* retinoic acid applications. Then, the cultures were treated with the selected concentration of the effective compounds for 24 h in 5% CO_2_. Total RNA was isolated using the PureLink RNA Mini Kit (Invitrogen, Grand Island, NY, USA) as described by the manufacturer. Briefly, the growth medium was removed from the cells, and 0.6 mL lysis buffer prepared with 2-mercaptoethanol was added to the sample. Then the lysate was transferred to 1.5 mL RNase-free tube and passed through an 18-21-gauge needle for 5–10 times. Following homogenization, the cell homogenate was washed with 70% ethanol and mixed thoroughly to disperse any visible residue. The sample was transferred to the spin cartridge (with the collection tube), centrifuged at 12,000× *g* for 15 s at room temperature. RNA was eluted from the membrane by adding 100 μL RNase–Free Water to the center of the Spin Cartridge. Finally, RNA yield and quality were determined using a plate reader (Multiskan, Thermo Labsystems, Vantaa, Finland) at wavelengths of 260 nm and 280 nm.

#### 2.10.2. cDNA Synthesis

Isolated total RNA was reverse transcribed into cDNA using High-Capacity cDNA Reverse Transcription kit (Applied Biosystems, Foster City, CA, USA) according to the provider’s manual. A 2× T master mix was prepared using kit components including 10× RT Buffer, 25× dNTP Mix (100 mM), 10× RT Random Primers, Reverse Transcriptase, and nuclease-free H_2_O. RNA samples were mixed with 2× RT master mix and loaded to thermal cycler (Sensoquest, Goettingen, Germany). The reaction was carried out at 25 °C for 10 min, at 37 °C for 120 min, then at 85 °C for 5 min. The cDNA was used directly for RT-PCR amplification or stored at −20 °C.

#### 2.10.3. PCR Array

The total cDNA was used in expression analysis via RT2 Profiler PCR Array Human Molecular Toxicology Pathway Finder (Qiagen, Valencia, CA, USA). To carry out the PCR array, 1150 μL SYBR Green PCR Master Mix (Applied Biosystems), 102 μL cDNA synthesis reaction, and 1048 μL RNase-free water was mixed. A 20 μL PCR components mix was added to each well of the RT2 Profiler PCR Array. Then, the RT2 Profiler PCR Array was sealed with Rotor-Disc Heat-Sealing Film using the Rotor-Disc Heat Sealer and loaded to real-time cycler (Qiagen Rotor-Gene Q). Triple repeated reactions were started with an initial denaturation step of 10 min at 95 °C, followed by 40 cycles of 15 s at 95 °C and 30 s at 60 °C. Melting curve analysis was utilized to validate the presence of a single PCR product. The threshold value was set above background signal, but within the lower one third to one half of the linear phase of the amplification plot. CT Cut-off value was selected as 35 cycle (program default) for the analyses. Data analysis was followed out with the ΔΔC_T_ method with normalization of the raw data to 5 different housekeeping genes, including beta-actin (*ACTB*), glyceraldehyde-3-phosphate dehydrogenase (*GAPDH*), heat shock protein 90 kDa alpha (*HSP90AB1*), phosphoglycerate kinase 1 (*PGK1*), and ribosomal protein lateral stalk subunit P0 (*RPLP0*). In the real time analysis, relative expressions were calculated using Livak (2^−ΔΔCt^) method that normalize the CT values according to the reference genes. CT values were used for analyzing fold changes of gene expression using PCR Array Data Analysis Software (The GeneGlobe Data Analysis Center, Qiagen) [25]. In the context of molecular genetic studies, 64 essential genes in 10 different biological pathways were evaluated.

### 2.11. Statistical Analyses

The data are presented as mean ± SD from at least five independent experiments. For statistical analyses, a one-way analysis of variance (ANOVA) and Tukey test was used using GraphPad Prism version 7. A *p*-value of less than 0.05 was considered as statistically significant.

## 3. Results and Discussion

### 3.1. Aβ_1-42_ Treatments Induced Cell Death in Differentiated SH-SY5Y Cells

**Figure 2:**
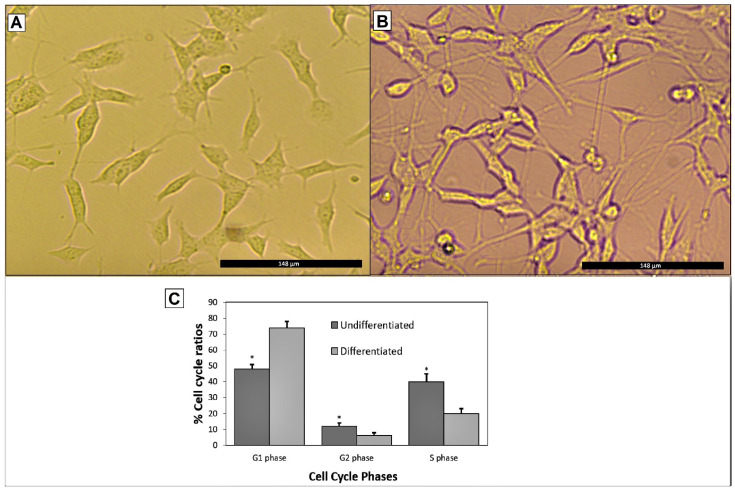
(**A**) Undifferentiated SHSY5Y cells, (**B**) differentiated SHSY5Y cells using a combination of RA+BDNF. (40× magnifications) and (**C**) cell cycle analysis via flow cytometry. Statistical analysis was performed using a one-way ANOVA followed by Tukey’s post-hoc test. Symbol (*) used for statistically significant (*p* < 0.05) change in each cell cycle phase.

SH-SY5Y human neuroblastoma cells’ differentiation process to neuronal-like cells included using a combination of RA and BDNF. According to the cell cycle analysis, total cells in the both S and G1 phases were significantly (*p* < 0.05) increased in differentiated SH-SY5Y cells compared to undifferentiated cell cultures (Figure 2). To choose the most appropriate *Aβ_1-42_* concentration for anti-AD assessments, a wide range of *Aβ* concentrations involving 1.25, 2.5, 5, 10, 20, 40, 80, 160, and 320 μM were applied into the cellular model for 24 h (n = 6). After exposing the SH-SY5Y cells with differentiation to *Aβ_1-42_*, cell viability rates were determined by MTT assay. The MTT assay results showed that *Aβ* induced toxicity in a concentration-dependent manner (Table 1). The highest concentration (320 μM) caused the sterility of the cultures. The lowest concentration (1.25 μM) induced cell death at a rate of 18.74% compared to control. The half-maximal inhibitory concentration (IC_50_) is generally used to measure drug potency in pharmacological research. A rate of 51.84% was calculated after exposure to 20 μM *Aβ_1-42_*; thus the protocol using the 24 h treatment of 20 μM *Aβ_1-42_* was selected for further experimental works [18]. 

### 3.2. Neuroprotective Potentials of Novel GPEs on Aβ_1-42_-Induced Cytotoxicity in In Vitro Cellular AD Model

The MTT and LDH cytotoxicity assays were used to investigate the effects of GPE, novel GPEs (as GPE1, GPE2 and GPE3), and MEM against *Aβ_1-42_* induced cytotoxicity. The cytotoxicity analyses were used to monitor membrane damage (LDH leakage) and mitochondrial function (MTT reduction) to calculate relative cell viability compared to negative control. The cultures without GPEs or MEM were assigned as negative control, and 1% Triton-X was used to stimulate positive control with *Aβ_1-42_* treatment. 

We determined statistically significant (*p* < 0.05) cell viability reductions at 20 μM of *Aβ_1-42_* treatment for 24 h. We obtained cell viability ratios of 52.73% and 44.28, in MTT and LDH, after *Aβ*-exposure. When different GPEs and MEM concentrations (0.1, 1, 10, 25, 50, and 100 μM) were used alone into the cell cultures, all concentrations (except 100 μM for MEM) did not alter the cell viability rates in comparison to untreated cells (*p* > 0.05). The observed cell viability rates after treatments with 100 μM MEM were 91.34% and 88.75% in MTT and LDH assays, respectively (Supplementary Figures S1 and S2). Based on this result, we revealed that the highest concentration of MEM would cause cytotoxicity on neuronal-like cells. In line with our finding, a previous report proved that MEM would influence intracellular pathways involved in cellular survival and/or apoptotic processes. Likewise, the cytotoxic action of 100 μM MEM was also recorded in cultured human peripheral blood and mouse N2a neuroblastoma cells [26]. 

**Figure 3:**
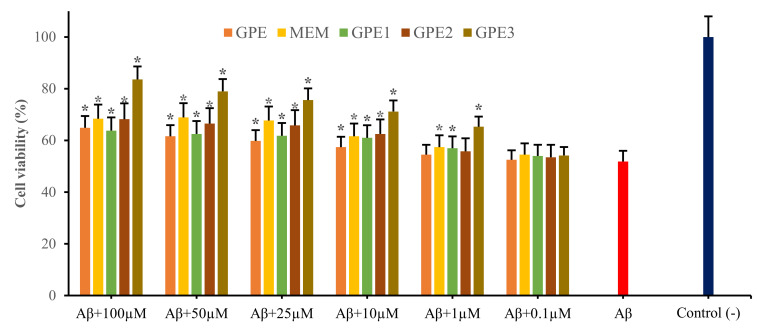
The neuroprotective effects of GPE, MEM, GPE1, GPE2, and GPE3 against in vitro *Aβ_1-42_*-exposure (MTT assay; % cell viability) (n = 5). Statistical analysis was performed using a one-way ANOVA followed by Tukey’s post-hoc test. Symbol (*) represents a statistically significant (*p* < 0.05) increase in cell viability compared to beta-amyloid.

**Figure 4:**
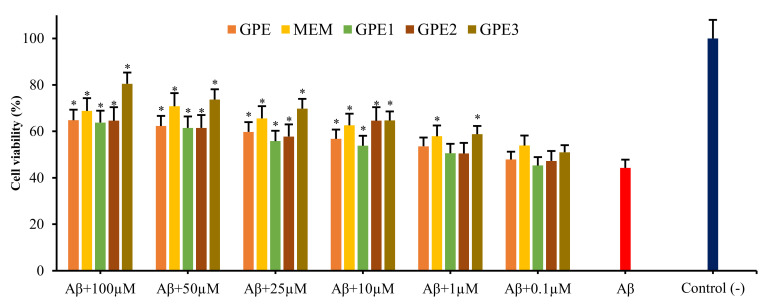
The neuroprotective effects of GPE, MEM, GPE1, GPE2, and GPE3 against in vitro *Aβ_1-42_*-exposure (LDH assay results converted to % of cell viabilities) (n = 5). Statistical analysis was performed using a one-way ANOVA followed by Tukey’s post-hoc test. Symbol (*) represents a statistically significant (*p* < 0.05) increase in cell viability compared to beta-amyloid.

MTT assay pointed out the significant inhibition of SH-SY5Y cell viability by *Aβ_1-42_* application (*p* < 0.05). Treatments with GPEs and MEM increased the number of viable cells significantly more than cells treated with *Aβ_1-42_* only. The neuroprotective action was associated with agent type and concentration. The addition of GPE3 at a various concentration rates tend to decrease cell damage induced by *Aβ_1-42_* than others (Figure 3). Similarly, results by LDH release assay were in accordance with those of MTT assay (Figure 4). A100 μM GPE3 showed the most protective effect. Moreover, cell viability analyses indicate a dose-dependent relationship between formulations and neuroprotective capabilities.

The MTT assay centers on the mitochondrial metabolic capacity of alive cells and the state of intracellular redox [27]. SH-SY5Y cells were treated with *Aβ_1-42_* for 24 h, and the *Aβ*-induced neurotoxicity was monitored via MTT assay. *Aβ* application led to a remarkable decrease in cell viability (20 μM; about 48%). Co-treatment with different concentrations of GPEs or MEM modulate *Aβ*-induced cytotoxicity. MTT testing revealed that differentiated SH-SY5Y cell cytotoxicity was minimized by co-treatments with GPEs or MEM concentrations. All tested formulations inhibited cell death induced by *Aβ*-exposure, resulting in a respective increase in the cell viability by 13.1, 12.0, 16.4, 31.8, and 16.6% for GPE, GPE1, GPE2, GPE3, and MEM at a concentration rate of 100 μM, in MTT assay. For evaluating the probability of *Aβ*-induced membrane damage, LDH assay was used to explore the protective effect of GPEs and MEM, measuring the activity of LDH released into the medium from necrotic and/or apoptotic SH-SY5Y cells. LDH assay fixed up an overlook for the percentage of surviving neuronal-like cells. With *Aβ_1-42_* treatment for 24 h, the released LDH enzyme by SH-SY5Y cells increased to a rate of 55.72% compared to negative control group. Present results denote that GPEs and MEM would protect the SH-SY5Y cell membrane against *Aβ*-induced neurotoxicity. Indeed, all GPEs and MEM inhibited cell death induced by *Aβ*-exposure, resulting in significant increases (*p* < 0.05) in the cell viability by 20.5, 19.5, 20.3, 41.7, and 24.5% for GPE, GPE1, GPE2, GPE3 and MEM treatments at a concentration of 100 μM, respectively, in LDH assay.

### 3.3. The In Vitro Effects of Novel GPEs on AChE Activity

**Figure 5:**
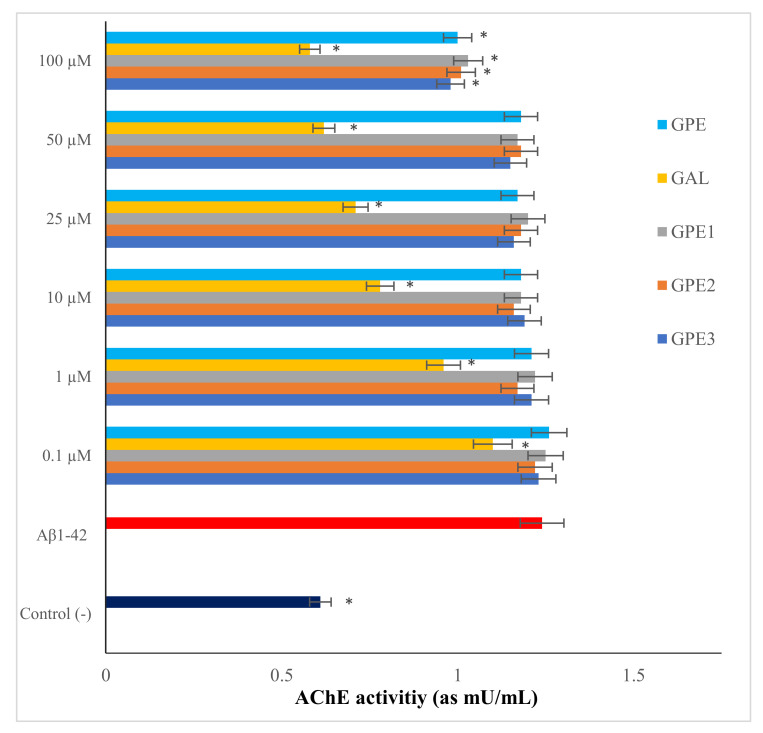
The effects of novel GPEs applications on *Aβ_1-42_*-induced AChE activity (n = 5). Statistical analysis was performed using a one-way ANOVA followed by Tukey’s post-hoc test. Symbol (*) represents a statistically significant (*p* < 0.05) decrease in AChE activity compared to beta-amyloid.

Recent transcriptional profiling data indicate that either undifferentiated or differentiated SH-SY5Y cells contain AChR mRNA and express nearly half of acetylcholine (ACh) receptor subunits [28]. Moreover, *Aβ_25-35_* or *Aβ_1-42_* led to significant increases in AChE expression in neuron-like human neuroblastoma cells [29]. Therefore, to find out the effects of GPEs on AChE activity in comparison to anticholinesterase drug, GAL, the differentiated SH-SY5Y cells were treated with GPEs and GAL or media alone for 24 h and then assessed for AChE activity (Figure 5). We found that 20 µM *Aβ_1-42_* approximately increased the baseline AChE activity by two-fold. However, treatment with GAL substantially regulated *Aβ*-induced AChE activity. However, treatments with GPEs provided such a slight modulation. In fact, GPE, GPE1, GPE2, and GPE3 (at only100 µM) could reduce *Aβ*-stimulated AChE activity at a rate of 19.35%, 16.93%, 18.54%, and 20.96% respectively (Figure 4). Present results clearly showed that all GPEs worked, but only at very high concentrations than GAL. At this point, several in vivo findings revealed that GPE has the potency to induce AChE released from cortical and striatal neuron cells, however the molecular justification of AChE released by GPE is still unclear [30]. The chemically modified GPE compound has been shown to have an opposite effect on AChE activity, due to modifications in its active groups. In this regard, the present results firstly indicate that GPEs would regulate the AChE activity via exhibiting a mild level of AChE inhibiting feature.

### 3.4. The In Vitro Effects of Novel GPEs on β-Secretase and α-Secretase Activities

Table 2 presents the results of measurement of α-secretase activity in differentiated SH-SY5Y cells treated with GPEs, *Aβ_1-42_*, or a combination of them. The GA application (at 50 µM, as positive control) concurrently led to a statistically (*p* < 0.05) significant increase in α-secretase activity, while reduced the activity of β-secretase in differentiated SHSY-5Y cell cultures. Similar to this finding, dual α- and β-secretase modulating effects by GA were also reported in clinical mouse model of AD [31]. Our analysis also clearly indicates that *Aβ_1-42_* treatment for 24 h would lead to a significant decrease (*p* < 0.05) of α-secretase activity compared to untreated cultures. Collaterally to this finding, the levels of α-secretase were found to be significantly lower in AD patients than in controls [32]. A previous report stated that when differentiated SH-SY5Y cells exposed to *Aβ_1-42_*, the activity of α-secretase was prominently decreased [33]. Therefore, α-secretase activation was generally considered to be of therapeutic value [34]. A 50 and 100 µM of GPE and GPE3 supported the increases of α-secretase activity against *Aβ_1-42_*-induced inactivation, however, the other GPEs were found to be ineffective in α-secretase activity. This is the first proof, which declares that certain GPE analogs would have a positive impact on α-secretase activity.

On the other hand, targeting *β*-secretase (BACE1) is also considered a very rational approach since this enzyme’s level is increased in the brain of patients with AD [35]. Similarly, the addition of *Aβ_1-42_* into the SH-SY5Y growth medium tend to increase *β*-secretase activity [36]. Following these previous reports, the present results indicate that *Aβ_1-42_* caused about 2.4-fold increases in *β*-secretase activity. At variance, the applications with different concentrations of GPEs did not alter *β*-secretase activity in cultured differentiated SH-SY5Y cells. However, the co-applications with novel GPEs could not reduce the increased level of *β*-secretase by *Aβ_1-42_* (Table 2). Thus, it is concluded that GPEs are not associated with *β*-secretase activity and amyloidogenic APP processing in vitro.

### 3.5. Effects of Applications with Novel GPEs on TAC and TOS Levels in the Cellular Model of AD

The effects of treatments with novel GPEs on TAC and TOS levels in the AD cellular model were evaluated (Table 3 and Table 4). The results revealed that all GPEs supported TAC levels. Indeed, the GPEs at 100 µM led to significant increases in TAC levels compared to control (-) cultures. GPE, GPE1, GPE2, GPE3, and ascorbic acid (10 µM, as positive control) increased TAC levels in about 2.37, 2.16, 1.97, 2,51- and 2.75-fold changes. On the contrary, only H_2_O_2_ (25 µM, as positive control) treatment caused significant increases (approximately 2.9-fold) of TOS levels. However, treatments with all GPEs did not alter the measured TOS levels compared to untreated controls (*p* > 0.05). The decreasing order of supporting antioxidant capacity by compounds were as GPE3 > GPE > GPE1 > GPE2. The obtained results also revealed that *Aβ_1-42_* exposure caused significant (*P* < 0.05) decreases of TAC and increases of TOS levels in vitro. In the same trend, 20 µM *Aβ_1-42_* treatment reduced TAC level by about 0.6-fold and enhanced TOS level about 2.3-fold. On the contrary, these negative alterations were modulated by GPE and its novel analogs co-treatment with *Aβ_1-42_.* GPE3 was more effective than other tested GPEs in alleviating oxidative stress by *Aβ_1-42_*-exposure (Table 3 and Table 4).

Following these present findings, it was reported that *Aβ_1-42_* increased the vulnerability of SH-SY5Y cells to oxidative stress [37]. It was evidenced that *Aβ_1-42_* initiated lipid peroxidation via inserting as oligomers into the bilayer and serving as a ROS source [38]. In addition to above mentioned in vitro studies, *Aβ_1-42_* treatments led to increase of superoxide dismutase (SOD) and catalase (CAT) enzyme activities, decrease of glutathione peroxidase (GSH-Px) activity and the total content of the reduced glutathione (GSH) and enhancement of malondialdehyde (MDA) and protein carbonyl levels in the hippocampus regions of rats [39]. Likewise, application with *Aβ_1-42_* generated ROS, 3-nitrotyrosine (3-NT), 4-hydroxy-nonenal (4-HNE), and 8-hydroxy-2’-deoxyguanosine (8-OH-dG) products in the mouse model of AD [40,41].

In the light of the present results, GPEs are shown to execute different degrees of antioxidative potential without leading pro-oxidative alterations. Moreover, co-treatment with GPEs decreased TOS and increased TAC levels compared with the *Aβ_1-42_*-treated group. These results propose that GPEs play a protective role against oxidative stress, which indicates that the antioxidant effects of GPEs are involved in the neuroprotection mechanism. Concordantly, this study’s results clearly revealed that novel GPEs propounded high antioxidative potency for the first time. It is well known that AD is a progressive neurodegenerative disease in aging brains and asserts a long and relatively asymptomatic prodromal phase. There is still no exact and efficient treatment option for AD [42]. Therapeutic approaches targeting the triggers (oxidative stress, neuronal apoptosis, etc.) of AD are being considered to produce the most significant advantage if applicated during the prodromal phase or earlier [43]. In this regard, GPEs might slow the progression or delay the onset of AD.

### 3.6. Effects of Novels GPEs on Apoptosis and Necrosis in the Cellular Model of AD

**Figure 6:**
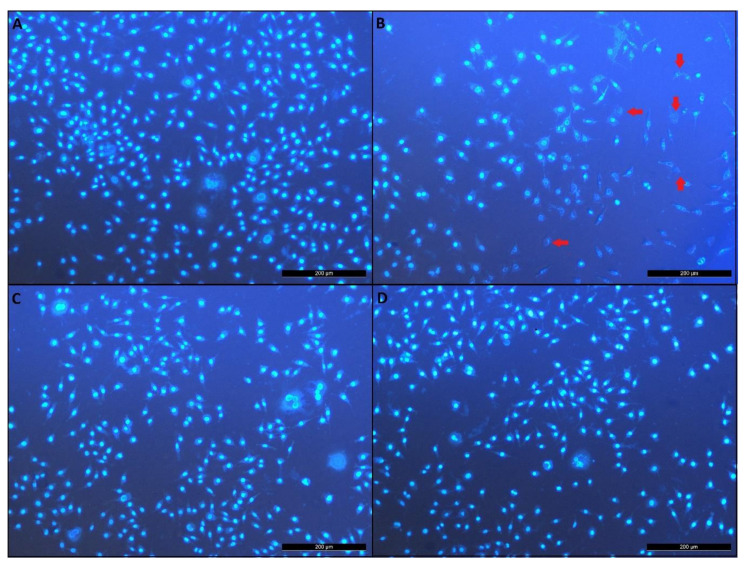
Effects of GPE and GPE3 on apoptosis and necrosis in cellular model of AD (Hoechst 33258) (n = 5), (**A**) Untreated group, (**B**) *Aβ_1-42_* (20 µM), (**C**) GPE3 (50 µM) + *Aβ_1-42_*, (**D**) GPE (50 µM) + *Aβ_1-42_.* Red arrows indicate necrotic cells with damaged chromosomal structures.

**Figure 7:**
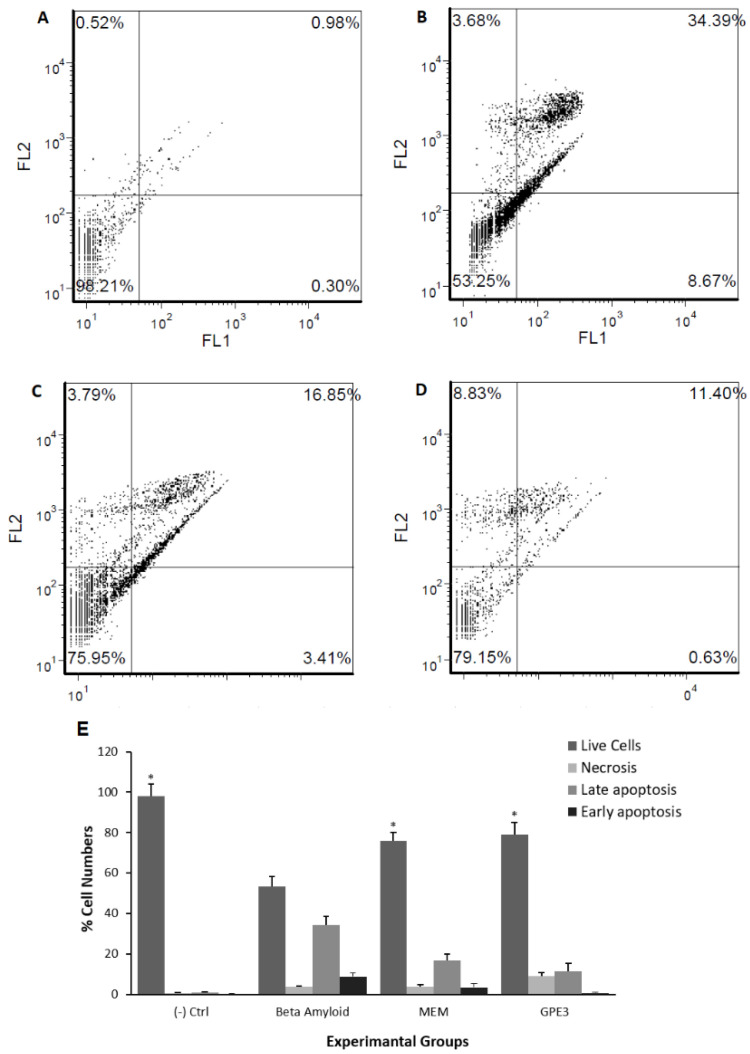
Flow cytometric analysis of Annexin V-FITC (FL1)/PI (FL2) double-labeled cells in a cellular model of AD (n = 5), (**A**) negative control, (**B**) *Aβ_1-42_*, (**C**) MEM (50 µM) + *Aβ_1-42_*, (**D**) GPE3 (50 µM) + *Aβ_1-42_*, (**E**) All experimental groups were shown in one graph. Statistical analysis was performed using a one-way ANOVA followed by Tukey’s post-hoc test. Symbol (*) represents a statistically significant (*p* < 0.05) increase in cell viability compared to beta-amyloid application.

Staining with Hoechst 33258 and visual examination revealed that a co-treatment with GPEs inhibited apoptosis by *Aβ_1-42_* at different levels (Figure 6A–D). The visual observations using apoptosis-necrosis assay indicated that *Aβ_1-42_* toxicity occurred via a necrotic or late apoptotic rather than the early apoptotic condition because of their smear-looking nucleus as indicated in several reports [44]. This assay demonstrated that GPE3 provided more protection potential from *Aβ_1-42_*-induced necrosis and late apoptosis in a dose-dependent manner than other GPEs and MEM. These results were correlated and quantified in the flow cytometry analysis (Figure 7). Since MEM could exhibit slight cytotoxicity at 100 µM, all comparisons were made only at 50 µM concentrations. And the decreasing order of effectiveness of GPEs and MEM against *Aβ_1-42_*-induced apoptosis were assessed as GPE3 > MEM > GPE > GPE1 > GPE2 [45].

Due to the determined higher potential of neuroprotection (MTT, LDH assays, and Hoechst 33258 staining), AChE inhibition potential, and antioxidative power against *Aβ_1-42_*-induced neurotoxicity in cellular AD model, further flow cytometric analysis was continued with GPE3. The effectiveness of GPE3 against *Aβ_1-42_*-induced apoptosis and necrosis was determined in comparison to MEM using flow cytometry. Flow cytometric analysis of apoptosis and necrosis showed that *Aβ_1-42_* application caused a significant (*p* < 0.05) cell death rate (34.39%) via apoptosis and a slight death rate (3.68%) via necrosis. On the contrary, GPE3 and MEM decreased the apoptotic cell percentage compared to *Aβ_1-42_* application by %22.8 and %31.03, respectively. In brief, GPE3 was considered more able to protect the human neuron-like cells from *Aβ_1-42_*-induced necrosis and apoptosis than MEM (Figure 7A–D).

### 3.7. Molecular Genetic Responses to GPE3 in Cellular AD Model

From the above-detailed results, it is reasonably concluded that GPE3 is the best GPE analog showing neuroprotective action against *Aβ_1-42_* induced neurotoxicity in vitro. To determine the exact molecular mechanisms underlying this neuroprotection by GPE3, RT^2^ Profiler PCR Arrays were applied. For this aim, different total RNA samples from each treatment were characterized via the human inflammatory cytokines and receptors, and the percentage of detectable genes was calculated for each amount. In this case, the PCR array could detect individual genes, despite the expression of related gene-family members in the same RNA sample. The observed gene expression changes after treatment with *Aβ_1-42_* (20 µM) and GPE3 (50 µM) plus *Aβ_1-42_* (20 µM) in comparison to untreated cells are presented in Supporting information (Table 5). In the content of molecular genetic studies, 64 essential genes in 10 different biological pathways were evaluated and discussed.

The results of the PCR array indicated that *Aβ_1-42_* caused remarkable increases in the *CASP8*, *CASP9*, and *FASLG* expressions. Again, a slight increase of *AKT1* expression was observed after *Aβ_1-42_* exposure. On the contrary, *Aβ_1-42_* caused significant decreases in the levels of *BCL2* and *BCL2L1* genes. In parallel to these findings, *Aβ-* induced neuronal apoptosis resulted from increased *CASP8* activation [46]. And a previous study determined that *Aβ* (as *Aβ_1-42_* and *Aβ_25-35_*) weakly activated *AKT1* in the SH-SY5Y neuroblastoma cell line [47]. On the other hand, *BCL2* and *BCL2L1*, anti-apoptotic genes, were reported to down-regulated by applying *Aβ_1-42_* in cultured rat hippocampal neurons and humans IMR-32 neuroblastoma cell line [48]. Besides, *FASLG* expression was significantly increased in senile plaques and neuro-filament-positive dystrophic neuritis, and relation to caspase activation and neuritic apoptosis in AD brain [49]. The present results also proved that treatment with GPE3 alone did not lead to significant expressional alterations on apoptosis or necrosis-related genes. However, GPE3 modulated the expressional alteration of specific genes involving *AKT1*, *BCL2*, *BCL2L*, *CASP8*, *CASP9*, and *FASLG* genes by *Aβ_1-42_*, and it was concluded that GPE related neuroprotection via anti-apoptotic and anti-necrotic effectiveness is based on the expression profiling of these genes. 

It was revealed that the elevation of *BRCA1* is a part of GPE3 related in vitro neuroprotection against neurotoxicity of *Aβ*. Again, *Aβ_1-42_* led to a significant increase in *DNAJB1* and *HSPA1A* activities. In parallel to the present results, the expressions of *DNAJB1* and *HSPA1A* genes were detected as up-regulated in patients with AD [50,51]. The present findings also revealed that GPE3 modulated the expressional change of the gene *HSPA1A* induced by *Aβ_1-42_*, and reduction of *HSPA1A* contributed to GPE3 related in vitro neuroprotection against neurotoxicity of *Aβ*. *Aβ_1-42_* also suppressed the expression of *HMOX1, NQO1,* and *SLC7A11* activities. Following present findings, pharmacological induction or genetic over-expression of *HMOX1* significantly ameliorated the neurotoxic effects of *Aβ_1-42_* in SH-SY5Y cells [52]. Likewise, free radical scavengers stimulated the Nrf2-dependent defensive gene *NQO1* in SH-SY5Y cells [53]. Contrary to present findings, microarray analysis determined the up-regulation of *SLC7A11* in patients with AD [54]. GPE3 treatment alone only provided a small contribution to *HMOX1* (0.68-fold change). Moreover, GPE3 modulated the expressions of the gene *HMOX1, NQO1,* and *SLC7A11* induced by *Aβ_1-42_*. Consequently, the elevation of reduced *HMOX1*, *NQO1*, and *SLC7A11* expressions were conferred as a backdrop of antioxidative protection by GPE3. 

The results of RT^2^ PCR array established that *Aβ_1-42_* significantly reduced the expressions of *ADM2*, *DNAJB9*, and *UHRF1* genes. Consonant with these obtained results, the downregulation of the *ADM2* gene was observed in neural progenitor cells (NPCs) derived from familial AD mutant *PSEN1* subjects [55]. *DNAJB9* suppressed the early stages of *Aβ_1-42_* aggregation in vitro. *UHRF1* was thought to be responsible for controlling cellular proliferation and differentiation under physiological conditions [56]. The present data also showed that treatment with GPE3 alone did not lead to any statistically significant (*p* > 0.05) expressional change of these genes. Further, GPE3 elevated the expressional change of the genes involving *ADM2*, *DNAJB9*, and *UHRF1* reduced by *Aβ_1-42_*. Thus, it was concluded that remarkable enhancement of *ADM2*, *DNAJB9*, and *UHRF1* made a significant contribution GPE3-stimulated in vitro neuroprotection against *Aβ* toxicity.

The experiences from animal studies suggested strong relationships between AD-associated abnormalities and brain fatty acid metabolism. AD-related pathologies inhibited homeostatic and regenerative functions of neural stem cells via perturbation of fatty acid metabolism [57]. The *ACADM* gene is responsible for producing medium-chain acyl-CoA dehydrogenase (MCAD) enzyme. Similarly, *ACADVL* is accountable for producing a very long-chain acyl-CoA dehydrogenase (VLCAD) enzyme. Both enzymes play a critical function in mitochondria. They are essential for fatty acid oxidation, which is the multistage procedure that metabolizes fats to energy. Additionally, *ACOX1* gene coded peroxisomal straight-chain acyl-CoA oxidase enzyme, and this enzyme also has a function in β-oxidation [58]. The PCR array analysis indicates that *Aβ_1-42_* significantly decrease *ACADM* and *ACADVL* gene expressions. GPE3 modulated the expression changes of *ACADVL* gene altered by *Aβ_1-42_*. 

*Aβ_1-42_* led to decreases in *METAP2* and *MK167* activities. *METAP2* is a cytoplasmic enzyme responsible for promoting cell proliferation and it was found in higher levels in SH-SY5Y neuroblastoma cells. MK167 is a reliable marker for assessing cell proliferation. It is involved in the pathogenesis of neurofibrillary degeneration in AD. Furthermore, GPE3 elevated the *METAP2* and *MK167* expressions that were reduced by *Aβ_1-42_*. It was concluded that GPE3 also protected against the neurotoxicity of *Aβ* by immune-related pathway. The molecular genetic analysis indicated that *Aβ_1-42_* treatment would reduce only *CYP2D6* gene expression and GPE3 would modulate changes in gene expression of *CYP2D6* altered by *Aβ_1-42_*. *CYP2D6* was shown to produce a toxin-metabolizing enzyme in SH-SY5Y cells and inactivates several neurotoxic substances. 

## 4. Conclusions

Herein, we developed novel anti-Alzheimer formulations, based on GPE1, GPE2, and GPE3. The present in vitro results firstly suggested that treatments with novel GPE analogs might be promising for treating or preventing AD. Their multimodal action potential could modulate oxidative stress, ACh depletion, α-secretase inactivation, apoptotic and necrotic cell death as well as immunosuppression, the principal hallmarks of AD. Taking together all these multiple activities associated with pathological conditions of AD, the present novel GPEs seem to be potential multifunctional candidate for further experimental works to develop new anti-Alzheimer agents. These experiments should be performed on AD animal models to get a more logical result to understand the main keys behind the neuroprotective mechanisms of GPE derivatives. Moreover, β-amyloid peptide fibrilization analyses can be performed to obtain supporting information from the aspect of time dependent toxicity to strengthen the knowledge regarding the mechanism behind neuroprotective properties of GPE1, GPE2, and GPE3 tripeptides.

## Figures and Tables

**Table 1:** Cytotoxicity of *Aβ_1-42_* on differentiated SH-SY5Y cells.

Group		Cell Viability (as %)
Control		100
*Aβ_1-42_* concentrations (as μM)	1.25	81.26 ± 5.06
	2.5	73.42 ± 5.24
	5	66.93 ± 6.55
	10	61.41 ± 4.87
	20	51.84 ± 5.21
	40	43.65 ± 4.38
	80	32.11 ± 4.22
	160	11.22 ± 2.78

**Table 2:** The effects of GPEs on α-secretase and β-secretase activities (as fluorescence intensity/100 µg protein). Statistical analysis was performed using a one-way ANOVA followed by Tukey’s post-hoc test. Means (n = 5) with the same letter in the same column are not significantly different at *p* = 0.05.

Group	α-Secretase Activity	β-Secretase Activity
Control (-)	18.8 ± 3.6 ^c^	2854.5 ± 308.6 ^b^
Control (+)	27.6 ± 4.7 ^d^	1635.6 ± 211.8 ^a^
20 µM *Aβ_1-42_*	6.2 ± 1.3 ^a^	6884.8 ± 545.0 ^c^
*Aβ* +0.1 µM GPE	6.1 ± 1.1 ^a^	6774.5 ± 478.2 ^c^
*Aβ* +1 µM GPE	6.4 ± 1.0 ^a^	6841.6 ± 396.5 ^c^
*Aβ* +10 µM GPE	6.8 ± 1.2 ^a^	6692.0 ± 383.4 ^c^
*Aβ* +25 µM GPE	7.4 ± 1.4 ^a^	6735.5 ± 411.7 ^c^
*Aβ* +50 µM GPE	10.6 ± 1.4 ^b^	6689.2 ± 549.4 ^c^
*Aβ* +100 µM GPE	12.5 ± 1.7 ^b^	6714.4 ± 661.0 ^c^
*Aβ* +0.1 µM GPE1	6.0 ± 0.8 ^a^	7028.7 ± 486.6 ^c^
*Aβ* +1 µM GPE1	6.3 ± 1.1 ^a^	6894.4 ± 463.1 ^c^
*Aβ* +10 µM GPE1	6.7 ± 1.0 ^a^	6838.5 ± 471.4 ^c^
*Aβ* +25 µM GPE1	6.9 ± 1.4 ^a^	6845.4 ± 504.5 ^c^
*Aβ* +50 µM GPE1	7.1 ± 1.2 ^a^	6771.2 ± 522.2 ^c^
*Aβ* +100 µM GPE1	7.4 ± 1.4 ^a^	6768.4 ± 478.2 ^c^
*Aβ* +0.1 µM GPE2	6.1 ± 1.0 ^a^	7041.4 ± 506.0 ^c^
*Aβ* +1 µM GPE2	6.4 ± 1.1 ^a^	7012.0 ± 543.6 ^c^
*Aβ* +10 µM GPE2	6.4 ± 1.3 ^a^	6861.4 ± 431.5 ^c^
*Aβ* +25 µM GPE2	6.7 ± 1.4 ^a^	6844.5 ± 447.7 ^c^
*Aβ* +50 µM GPE2	7.0 ± 1.5 ^a^	6864.2 ± 391.2 ^c^
*Aβ* +100 µM GPE2	7.2 ± 1.3 ^a^	6785.0 ± 488.0 ^c^
*Aβ* +0.1 µM GPE3	6.1 ± 1.7 ^a^	6993.0 ± 512.6 ^c^
*Aβ* +1 µM GPE3	6.4 ± 1.3 ^a^	6981.4 ± 455.2 ^c^
*Aβ* +10 µM GPE3	6.9 ± 1.5 ^a^	6873.5 ± 476.6 ^c^
*Aβ* +25 µM GPE3	7.3 ± 1.3 ^a^	6865.6 ± 442.0 ^c^
*Aβ* +50 µM GPE3	10.5 ± 1.6 ^b^	6756.2 ± 465.6 ^c^
*Aβ* +100 µM GPE3	12.8 ± 1.8 ^b^	6776.0 ± 511.8 ^c^

**Table 3:** The effects of GPEs on TAC and TOS levels in cultured differentiated SH-SY5Y cells. Statistical analysis was performed using one-way ANOVA followed by Tukey’s post-hoc test. Means (n = 5) with the same letter in the same column are not significantly different at *p* = 0.05.

Group		TAC Level (mmolTrolox Equiv./L)	TOS Level (µmol H_2_O_2_ Equiv./L)
Control (-)		5.08 ± 0.62 ^a^	1.86 ± 0.24 ^a^
Control (+)		13.96 ± 1.14 ^e^	5.45 ± 0.42 ^b^
GPE	0.1 µM	5.22 ± 0.41 ^a^	1.77 ± 0.27 ^a^
	1 µM	5.49 ± 0.57 ^a^	1.81 ± 0.21 ^a^
	10 µM	6.38 ± 0.48 ^b^	1.88 ± 0.14 ^a^
	25 µM	8.69 ± 0.63 ^bc^	1.83 ± 0.20 ^a^
	50 µM	10.91 ± 0.57 ^c^	1.87 ± 0.22 ^a^
	100 µM	12.08 ± 0.96 ^c^	1.90 ± 0.18 ^a^
GPE1	0.1 µM	5.06 ± 0.35 ^a^	1.84 ± 0.20 ^a^
	1 µM	5.38 ± 0.51 ^a^	1.73 ± 0.16 ^a^
	10 µM	6.79 ± 0.39 ^b^	1.82 ± 0.18 ^a^
	25 µM	8.25 ± 0.70 ^bc^	1.68 ± 0.20 ^a^
	50 µM	9.78 ± 0.61 ^c^	1.83 ± 0.22 ^a^
	100 µM	11.02 ± 0.94 ^c^	1.95 ± 0.22 ^a^
GPE2	0.1 µM	5.08 ± 0.40 ^a^	1.73 ± 0.26 ^a^
	1 µM	5.21 ± 0.61 ^a^	1.85 ± 0.21 ^a^
	10 µM	5.93 ± 0.71 ^a^	1.91 ± 0.26 ^a^
	25 µM	7.44 ± 0.58 ^b^	1.87 ± 0.18 ^a^
	50 µM	8.68 ± 0.56 ^bc^	1.93 ± 0.20 ^a^
	100 µM	10.04 ± 0.83 ^c^	1.98 ± 0.28 ^a^
GPE3	0.1 µM	5.25 ± 0.30 ^a^	1.66 ± 0.22 ^a^
	1 µM	5.86 ± 0.43 ^a^	1.64 ± 0.18 ^a^
	10 µM	7.73 ± 0.54 ^b^	1.74 ± 0.16 ^a^
	25 µM	9.18 ± 0.66 ^c^	1.88 ± 0.25 ^a^
	50 µM	10.94 ± 0.64 ^c^	1.81 ± 0.21 ^a^
	100 µM	12.74 ± 0.87 ^d^	1.89 ± 0.17 ^a^

**Table 4:** The effects of GPEs on TAC and TOS levels in a cellular experimental model of AD (n = 5). Statistical analysis was performed using one-way ANOVA followed by Tukey’s post-hoc test. Means with the same letter in the same column are not significantly different at *p* = 0.05.

Groups	TAC Level (mmolTrolox Equiv./L)	TOS Level (µmol H_2_O_2_ Equiv./L)
Control (-)	5.08 ± 0.62 ^b^	1.86 ± 0.24 ^a^
Control (+)	13.96 ± 1.14 ^f^	5.45 ± 0.42 ^d^
20 µM *Aβ_1-42_*	3.22 ± 0.58 ^a^	4.37 ± 0.40 ^c^
*Aβ* +0.1 µM GPE	3.89 ± 0.46 ^a^	4.09 ± 0.36 ^bc^
*Aβ* +1 µM GPE	4.95 ± 0.61 ^b^	3.85 ± 0.34 ^b^
*Aβ* +10 µM GPE	6.24 ± 0.54 ^bc^	3.27 ± 0.40 ^b^
*Aβ* +25 µM GPE	7.12 ± 0.63 ^c^	2.89 ± 0.33 ^b^
*Aβ* +50 µM GPE	9.65 ± 0.72 ^d^	2.60 ± 0.20 ^b^
*Aβ* +100 µM GPE	10.04 ± 0.79 ^d^	2.34 ± 0.28 ^b^
*Aβ* +0.1 µM GPE1	3.41 ± 0.52 ^a^	4.13 ± 0.26 ^c^
*Aβ* +1 µM GPE1	4.35 ± 0.54 ^ab^	3.96 ± 0.32 ^bc^
*Aβ* +10 µM GPE1	5.12 ± 0.47 ^b^	3.63 ± 0.34 ^b^
*Aβ* +25 µM GPE1	5.34 ± 0.45 ^b^	3.17 ± 0.35 ^b^
*Aβ* +50 µM GPE1	5.71 ± 0.50 ^b^	2.87 ± 0.21 ^b^
*Aβ* +100 µM GPE1	6.33 ± 0.55 ^bc^	2.66 ± 0.23 ^b^
*Aβ* +0.1 µM GPE2	3.88 ± 0.42 ^a^	4.02 ± 0.30 ^bc^
*Aβ* +1 µM GPE2	4.22 ± 0.51 ^a^	3.84 ± 0.42 ^b^
*Aβ* +10 µM GPE2	4.41 ± 0.39 ^a^	3.56 ± 0.33 ^b^
*Aβ* +25 µM GPE2	4.49 ± 0.53 ^a^	3.29 ± 0.25 ^b^
*Aβ* +50 µM GPE2	4.83 ± 0.51 ^b^	3.08 ± 0.26 ^b^
*Aβ* +100 µM GPE2	6.38 ± 0.55 ^bc^	2.93 ± 0.30 ^b^
*Aβ* +0.1 µM GPE3	4.15 ± 0.35 ^a^	4.06 ± 0.28 ^bc^
*Aβ* +1 µM GPE3	4.78 ± 0.47 ^b^	3.66 ± 0.36 ^b^
*Aβ* +10 µM GPE3	5.34 ± 0.45 ^b^	3.15 ± 0.27 ^b^
*Aβ* +25 µM GPE3	7.66 ± 0.81 ^c^	2.71 ± 0.32 ^ab^
*Aβ* +50 µM GPE3	9.86 ± 0.73 ^d^	2.55 ± 0.18 ^ab^
*Aβ* +100 µM GPE3	10.93 ± 0.88 ^e^	2.17 ± 0.26 ^a^

**Table 5:** The gene expression alterations (as fold change).

Gene	*Aβ _1-42_*	*Aβ _1-42_* Plus GPE3
*ACADVL*	−0.71 ± 0.05	5.65 ± 1.25
*ADM2*	−0.38 ± 0.12	2.90 ± 0.28
*AKT1*	0.15 ± 0.02	0.09 ± 0.03
*BCL2*	−0.86 ± 0.07	0.34 ± 0.01
*BCL2L1*	−0.91 ± 0.11	0.72 ± 0.03
*BRCA1*	−0.18 ± 0.02	6.92 ± 0.48
*CASP8*	3.27 ± 0.21	0.29 ± 0.02
*CASP9*	2.87 ± 0.35	0.54 ± 0.05
*CYP2D6*	−0.22 ± 0.21	3.94 ± 0.16
*DNAJB9*	−0.83 ± 0.18	16.95 ± 1.80
*FASLG*	0.94 ± 0.04	0.35 ± 0.03
*HMOX1*	−0.47 ± 0.12	8.95 ± 1.55
*HSPA1A*	3.80 ± 0.21	1.15 ± 0.45
*METAP2*	−0.66 ± 0.03	3.35 ± 0.90
*MK167*	−0.26 ± 0.01	3.20 ± 0.84
*NQO1*	−0.65 ± 0.06	5.66 ± 1.02
*SLC7A11*	−0.33 ± 0.02	6.96 ± 2.05
*UHRF1*	−0.81 ± 0.05	8.55 ± 1.60

## Data Availability

The data presented in this study are available on request from the corresponding authors.

## Supplementary Materials

The following are available online at https://www.mdpi.com/2218-273X/11/1/126/s1, Figure S1: The effects of GPE, MEM, GPE1, GPE2 and GPE3 on cell viability rates in differentiated SHSY5Y cells (MTT assay; % Cell viability). Symbol (*) represents statistically significant (*p* < 0.05) decreasing cell viability as compared to negative control (control-) group, Figure S2: The effects of GPE, MEM, GPE1, GPE2 and GPE3 on cell viability rates in differentiated SHSY5Y cells (LDH assay; % Cell viability). Symbol (*) represents statistically significant (*p* < 0.05) decrease in cell viability as compared to negative control (control-) group.
