# Supplementary material for: Glycyl-L-Prolyl-L-Glutamate Pseudotripeptides for Treatment of Alzheimer’s Disease
Source: Biomolecules. 2021 Jan 19;11(1):126. doi: 10.3390/biom11010126 (PMC7835747; doi:10.3390/biom11010126)
Supplement: Supplementary file 1 [file biomolecules-11-00126-s001.pdf]

Supplementary Materials

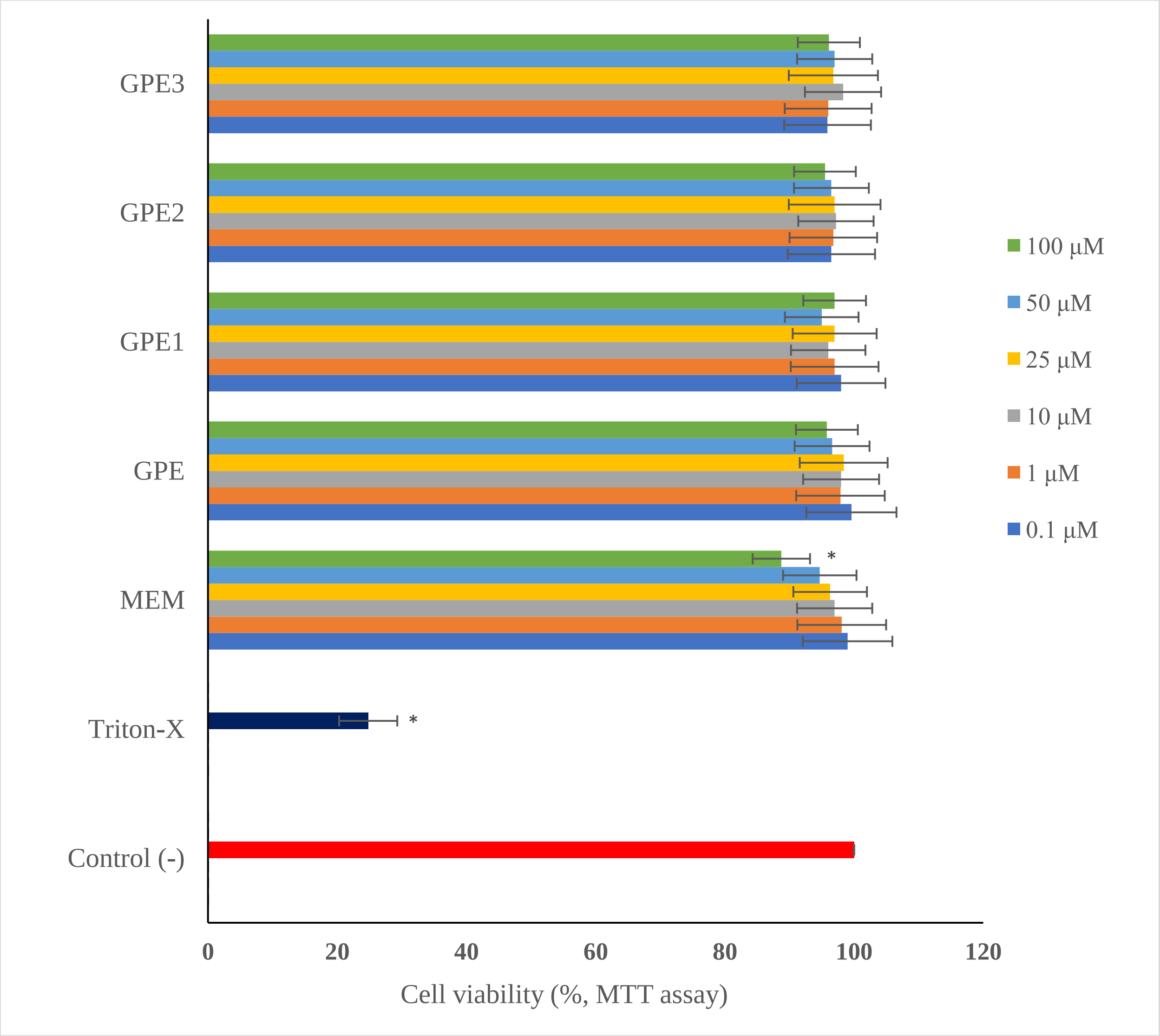

Figure 1. The effects of GPE, MEM, GPE1, GPE2 and GPE3 on cell viability rates in differentiated SHSY5Y cells (MTT assay; % Cell viability). Symbol (*) represents statistically significant ($p<0.05$) decrease in cell viability as compared to negative control (control-) group.

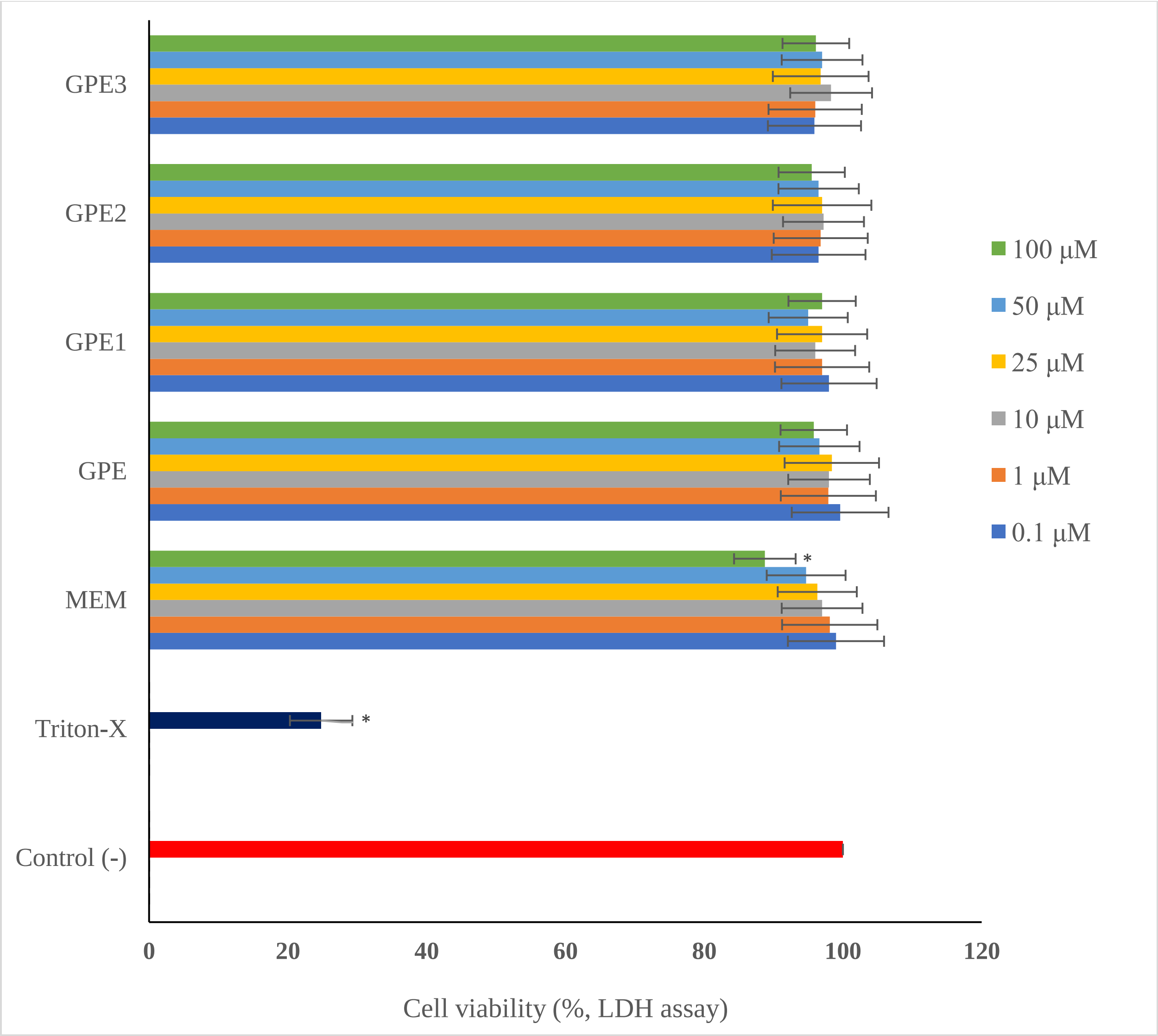

Figure S2. The effects of GPE, MEM, GPE1, GPE2 and GPE3 on cell viability rates in differentiated SHSY5Y cells (LDH assay; % Cell viability). Symbol (*) represents statistically significant ($p<0.05$) decrease in cell viability as compared to negative control (control-) group.
